# Effect of China national centralized drug procurement policy on anticoagulation selection and hemorrhage events in patients with AF in Suining

**DOI:** 10.3389/fphar.2024.1365142

**Published:** 2024-02-20

**Authors:** Qi Zhang, Ruili Wang, Lei Chen, Wensu Chen

**Affiliations:** ^1^ Suining County People’s Hospital, Suining, China; ^2^ The Affiliated Hospital of Xuzhou Medical University, Xuzhou, Jiangsu, China

**Keywords:** anticoagulants, non-valvular atrial fibrillation, atrial fibrillation, vitamin K anticoagulants, Chinese yuan

## Abstract

**Background:** Launched in March 2019, the National Centralized Drug Procurement (NCDP) initiative aimed to optimize the drug utilization framework in public healthcare facilities. Following the integration of Non-Vitamin K Antagonist Oral Anticoagulants (NOACs) into the procurement catalog, healthcare establishments in Suining swiftly transitioned to the widespread adoption of NOACs, beginning 1 March 2020.

**Objective:** This study aims to comprehensively assess the impact of the NCDP policy on the efficacy of anticoagulation therapy, patient medication adherence, and the incidence of hemorrhagic events in individuals with non-valvular atrial fibrillation (NVAF) residing in Suining. The analysis seeks to elucidate the broader impacts of the NCDP policy on this patient demographic.

**Methods:** This study analyzed patient hospitalization records from the Department of Cardiology at Suining County People’s Hospital, spanning 1 January 2017, to 30 June 2022. The dataset included demographic details (age, sex), type of health insurance, year of admission, hospitalization expenses, and comprehensive information on anticoagulant therapy utilization. The CHA_2_DS_2_-VASc scoring system, an established risk assessment tool, was used to evaluate stroke risk in NVAF patients. Patients with a CHA2DS2-VASc score of 2 or higher were categorized as high-risk, while those with scores below 2 were considered medium or low-risk.

**Results:** 1. *Treatment Cost Analysis*: The study included 3,986 patients diagnosed with NVAF. Following the implementation of the NCDP policy, a significant increase in the average treatment cost for hospitalized patients was observed, rising from 8,900.57 ± 9,023.02 CNY to 9,829.99 ± 10,886.87 CNY (*p* < 0.001). 2. *Oral Anticoagulant Utilization*: Overall, oral anticoagulant use increased from 40.02% to 61.33% post-NCDP (*p* < 0.001). Specifically, NOAC utilization among patients dramatically rose from 15.41% to 90.99% (*p* < 0.001). 3. *Hemorrhagic Events*: There was a significant decrease in hemorrhagic events following the NCDP policy, from 1.88% to 0.66% (*p* = 0.01). Hypertension [*OR* = 1.979, 95% *CI* (1.132, 3.462), *p* = 0.017], history of stroke [*OR* = 1.375, 95% *CI* (1.023, 1.847), *p* = 0.035], age ≥65 years [*OR* = 0.339, 95% *CI* (0.188, 0.612), *p* < 0.001], combination therapy of anticoagulants and antiplatelets [*OR* = 3.620, 95% *CI* (1.752, 7.480), *p* < 0.001], hepatic and renal insufficiency [*OR* = 4.294, 95% *CI* (2.28, 8.084), *p* < 0.001], and the NCDP policy [*OR* = 0.295, 95% *CI* (0.115, 0.753), *p* = 0.011] are significant risk factors for bleeding in patients with atrial fibrillation. 4. *Re-hospitalization and Anticoagulant Use*: Among the 219 patients requiring re-hospitalization, there was a notable increase in anticoagulant usage post-NCDP, from 36.07% to 59.82% (*p* < 0.001). NOACs, in particular, saw a substantial rise in usage among these patients, from 11.39% to 80.92% (*p* < 0.001). 5. *Anticoagulant Type Change*: The NCDP policy [*OR* = 28.223, 95% *CI* (13.148, 60.585), *p* < 0.001] and bleeding events [*OR* = 27.772, 95% *CI* (3.213, 240.026), *p* = 0.003] were significant factors influencing the alteration of anticoagulant medications in patients.

**Conclusion:** The NCDP policy has markedly improved anticoagulation management in patients with AF. This policy has played a crucial role in enhancing medication adherence and significantly reducing the incidence of hemorrhagic events among these patients. Additionally, the NCDP policy has proven to be a key factor in guiding the selection and modification of anticoagulant therapies in the AF patient population.

## 1 Introduction

Atrial fibrillation (AF), a common clinical arrhythmia, significantly impacts not only patients but also their families and healthcare systems (Bekwelem et al., 2015; Kamath et al., 2021). Epidemiological studies reveal that AF’s prevalence among adults currently ranges from 2% to 4%. However, due to advancements in healthcare, increasing life expectancy, and improved AF detection (Staerk et al., 2017), the prevalence of AF is projected to increase by approximately 2.3 times (Colilla et al., 2013; Krijthe et al., 2013; Chugh et al., 2014). A study by the China Heart Study (CHS) from 2012 to 2015 found that the prevalence of AF in Chinese residents aged over 35 was 0.7%, with a higher occurrence in rural areas (0.75%) compared to urban areas (0.63%) (Wang et al., 2018).

In the realm of stroke prevention for AF patients, non-vitamin K antagonist oral anticoagulants (NOACs) and vitamin K anticoagulants (VKA) are predominantly used, especially among those newly initiating anticoagulation therapy (Heidbuchel et al., 2015). The China National Stroke Screening Program (CNSSS) survey from 2013 to 2014, which included 1,252,703 adults aged over 40 (Guo et al., 2018), indicated that 12% of ischemic stroke patients in China had concurrent AF. It is estimated that over 2.15 million ischemic stroke patients in China have AF. Alarmingly, only about 2.2% of these patients are on anticoagulation therapy, with VKA constituting 98.2% of these prescriptions.

The cost of medications is a critical determinant of overall treatment expenses, directly influencing drug accessibility and affordability (National Academies of Sciences E and Medicine, 2018). Research has consistently shown the efficacy of bulk purchasing in reducing the prices of drugs that are successfully included in procurement contracts (Tang et al., 2019; Long et al., 2022). Many countries have effectively implemented centralized drug procurement strategies, achieving notable reductions in drug prices and realizing substantial savings in pharmaceutical spending (Dylst et al., 2011; Seidman and Atun, 2017).

In an effort to refine the drug utilization system within public hospitals and tackle the ongoing challenges of limited healthcare access and medication adherence, China introduced the NCDP policy in March 2019. The overarching goal of this policy is to significantly enhance the procurement process, ensuring streamlined and efficient distribution of medications across the country.

As of August 2021, the NCDP policy in China has progressed through five phases, now encompassing an extensive list of 219 drugs, including new oral anticoagulants. This policy has been instrumental in significantly reducing the average price of the first batch of bid-winning products by 52%. Notably, the most substantial price reduction recorded was 96%. The implementation of the “4 + 7”policy has been a key factor in achieving these results, playing a pivotal role in lowering drug prices and promoting the rational use of medications (Chen et al., 2020).

While the current focus is predominantly on the NCDP policy’s role in reducing drug prices and expenditures in China, there is a notable gap in research concerning the policy’s impact at the patient level. It is crucial to redirect our focus to comprehensively understand the direct effects of this policy on patient outcomes. By undertaking detailed studies centered on individual patients, we can acquire critical insights into the NCDP policy’s efficacy in enhancing patient-oriented healthcare services.

Our study specifically aims to conduct a comparative analysis of various factors, including gender, age, incidence of bleeding events, types of anticoagulants used, and hospitalization costs incurred by AF patients before and after the implementation of the NCDP policy. By assessing these variables, our objective is to unravel the overarching impact of the NCDP policy on the management and treatment outcomes of AF patients at Suining County People’s Hospital. This approach is anticipated to provide a more nuanced understanding of how policy changes translate into real-world effects on patient care and health outcomes.

## 2 Materials and methods

### 2.1 Patient recruitment

**FIGURE 1:**
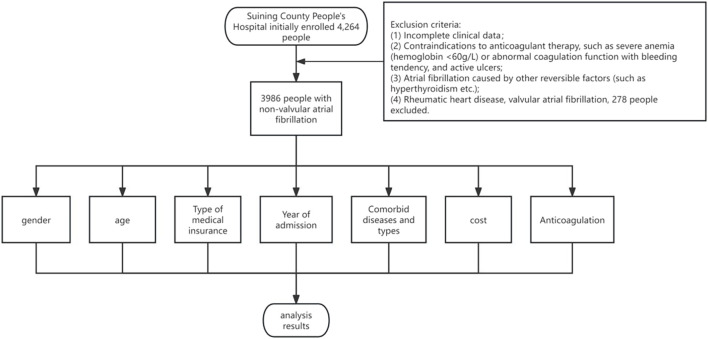
Research flow chart.

Patient Cohort and Study Criteria: Figure 1 presents the cohort of patients consecutively admitted with a diagnosis of NVAF at the Department of Cardiology, Suining County People’s Hospital, over the period from 1 January 2017, to 30 June 2022. The study meticulously collected and analyzed data pertaining to the general characteristics and clinical profiles of these patients. For patient inclusion, the criteria were as follows: (1) age ≥18 years, (2) a confirmed diagnosis of NVAF, in accordance with the 2020 guidelines set forth by the European Society of Cardiology (ESC) and the European Association for Cardio-Thoracic Surgery (EACTS) (Sepehri Shamloo et al., 2021).

Exclusion Criteria: (1) incomplete clinical data, (2) contraindications to anticoagulation therapy (e.g., severe anemia with hemoglobin levels below 60 g/L, abnormal coagulation profiles, bleeding tendencies, active ulcers, or any condition posing an elevated risk of bleeding complications), (3) AF diagnoses attributable to reversible factors like hyperthyroidism, (4) patients suffering from rheumatic heart disease or valvular AF. The initial assessment encompassed 4,264 individuals for potential inclusion. Following the application of these criteria, 278 individuals were excluded, culminating in a final cohort of 3,986 participants for the study.

### 2.2 Information collecting

The study conducted a thorough collection of patient data, encompassing key demographic information such as age, gender, and the type of medical insurance coverage. In addition to these basic parameters, the research extended to gathering extensive clinical data. This included detailed information on the type and number of concurrent diseases, the CHA2DS2-VASc score (Lip et al., 2018), total hospitalization expenses, and the specific utilization patterns of anticoagulant medications. Based on previous studies (Schulman et al., 2005; Bahit et al., 2017), bleeding events were divided into various subtypes.

### 2.3 Statistical analysis

The collected data were subjected to statistical analysis using SPSS 22.0 software. Measurement data that followed a normal distribution were presented as mean ± standard deviation ( $\bar{x}$ ±s) and compared using an independent sample *t*-test. Categorical data were expressed as percentages (%) and analyzed using the Pearson *χ* ^2^ test. Univariate logistic regression analysis was conducted to identify factors influencing anticoagulation. *p* < 0.05 was considered statistically significant in all analyses.

## 3 Results

### 3.1 Descriptive analysis

The study included a total of 3,986 cases that met the inclusion criteria, spanning from 1 January 2017, to 30 June 2022. The mean age of the participants was 76.58 ± 11.24 years. As depicted in Table 1, the cohort comprised 2,010 males (50.43%) and 1,976 females (49.57%). Notably, 120 participants (3.01%) were without medical insurance, whereas a significant majority, totaling 3,866 (96.99%), were covered by medical insurance.

**TABLE 1:** Comparison of general data before and after NCDP.

Group	Before NCDP	After NCDP		*p*
Gender, n (%)				
Male	1521 (49.37)	455 (50.28)	*χ* ^2^ = 0.231	0.631
Female	1560 (50.63)	450 (49.72)		
Age (year)	77.23 ± 11.194	74.37 ± 11.133	*t =* 6.759	**0.000**
Expenses (CNY)	8900.57 ± 9023.02	9829.99 ± 10886.87	*t =* 2.342	**0.019**
Type of medical insurance, n (%)				
Non-medical insurance	99 (3.21)	21 (2.32)	*χ* ^2^ = 0.910	0.167
Medical insurance	2982 (96.79)	884 (97.68)		
CHA2DS2-VASc score (scores)	2.93 ± 1.43	3.17 ± 1.49	*t =* 4.405	**0.000**
Anticoagulants, n (%)				
Oral anticoagulant	1233 (40.02)	555 (61.33)	*χ* ^2^ = 71.183	**0.000**
No oral anticoagulant	1848 (59.98)	350 (38.67)		
Oral drug type, n (%)				
VKA	1043 (84.59)	50 (9.01)	*χ* ^2^ = 920.129	**0.000**
NOACs	190 (15.41)	505 (90.99)		

Abbreviations: VKA, vitamin K anticoagulants; NOACs, Non-vitamin K antagonist oral anticoagulants; NCDP, national centralized drug procurement; bold, *p* < 0.05).

### 3.2 Comparison of general data before and after NCDP

Gender Distribution and Hospitalization Costs: According to Table 1, prior to the NCDP policy, the hospitalized cohort consisted of 1,521 males (49.37%) and 1,560 females (50.63%). Post-policy implementation, these figures altered to 455 males (50.28%) and 450 females (49.72%). Statistical analysis revealed no significant difference in gender distribution before and after the NCDP policy enactment (*χ* ^2^ = 0.231, *p* > 0.05). The average hospitalization cost prior to the NCDP policy was 8,900.57 ± 9,023.02 CNY, increasing to 9,829.99 ± 10,886.87 CNY post-implementation. This increase in hospitalization costs was statistically significant (*t* = 2.342, *p* < 0.05).

Before the NCDP policy, 99 (3.21%) patients lacked medical insurance, compared to 21 (2.32%) after the policy’s implementation. The distribution of medical insurance types showed no significant difference pre and post-NCDP (*χ* ^2^ = 0.910, *p* > 0.05).

The average CHA2DS2-VASc score among patients was 2.93 ± 1.43 before NCDP and 3.17 ± 1.49 after. This difference was statistically significant(*t* = 4.405, *p* < 0.05). Regarding anticoagulant use, 1,233 of 3,081 patients (40.02%) hospitalized before NCDP was prescribed oral anticoagulants (VKA or NOACs), compared to 1,848 (59.98%) who did not receive anticoagulants. Specifically, 1,043 (84.59%) were on VKA and 190 (15.41%) on NOACs before NCDP. Post NCDP, of 905 hospitalized patients, 555 (61.33%) received anticoagulant therapy, with 50 (9.01%) on VKA and 505 (90.99%) on NOACs, while 350 (38.67%) did not receive any anticoagulants. Following the implementation of the NCDP, there was a marked increase in the prescription of oral anticoagulants, with a notable rise in NOACs usage and a corresponding decrease in VKA prescriptions, as evidenced by significant statistical values (*χ* ^2^ = 71.183、920.129, *p* < 0.001).

### 3.3 Analysis of the effect of antiplatelet therapy on anticoagulation before and after NCDP

**FIGURE 2:**
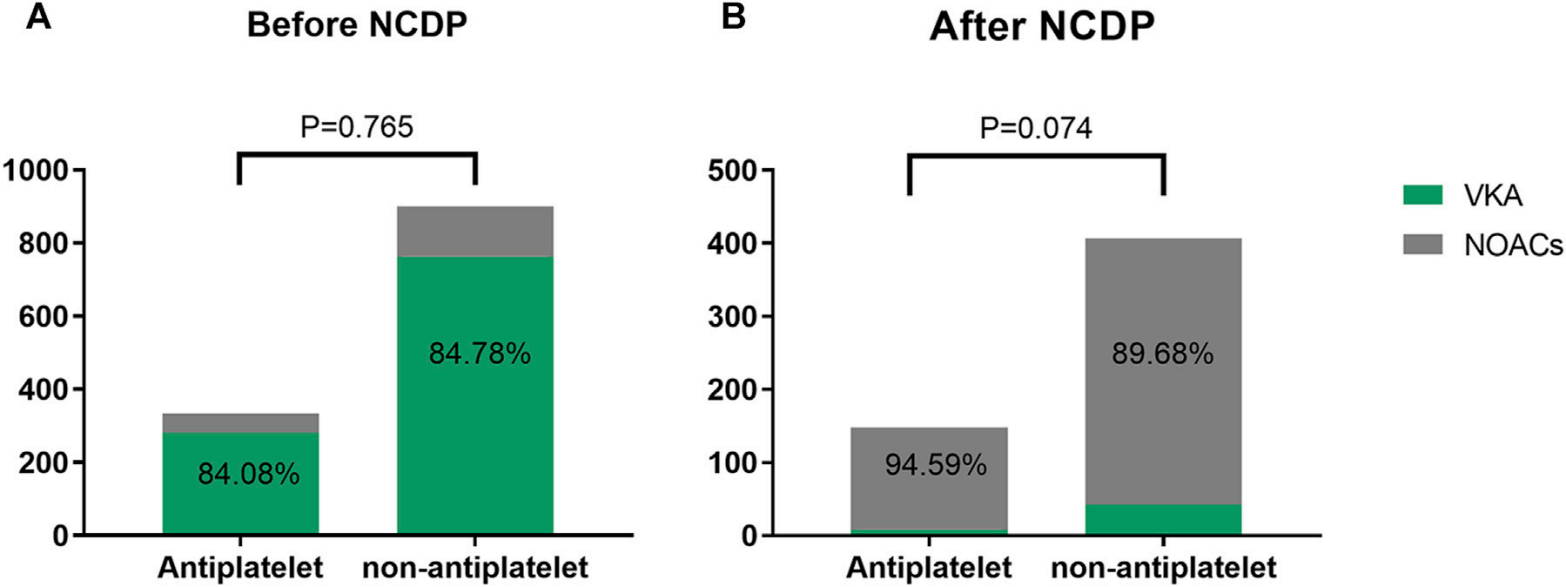
Analysis of the effect of antiplatelet therapy on anticoagulation before **(A)** and after NCDP **(B)**.

Figure 2 depicts the treatment patterns observed prior to the implementation of the NCDP policy. Among the study participants, 1,347 were administered antiplatelet therapy, with 333 of these individuals also receiving anticoagulation therapy. In the anticoagulation subgroup, 280 patients (84.08%) were treated with VKA, while 53 patients (15.92%) received oral NOACs. Of the study cohort, 1,734 patients did not undergo oral antiplatelet therapy. Among the 900 patients who were administered anticoagulant therapy, 763 (84.78%) were on VKA anticoagulants, and 137 (15.22%) were on NOACs.

Post-NCDP policy implementation, 301 patients were treated with antiplatelet therapy. Of these, 148 received anticoagulant therapy, with 8 (5.41%) being treated with VKA and 140 (94.59%) with NOACs. Additionally, 604 patients opted against oral antiplatelet therapy. Within this group, 407 received anticoagulant therapy, with 42 (10.32%) on VKA and 365 (89.68%) on NOACs. The choice of anticoagulant drugs, in relation to antiplatelet therapy, did not show significant change before and after the NCDP policy(*χ* ^2^ = 0.090、3.197, *p* > 0.05), as detailed in Table 2.

**TABLE 2:** The effect of antiplatelet therapy on anticoagulation before and after NCDP.

Group	Before NCDP		After NCDP	
	VKA, n (%)	NOACs, n (%)	VKA, n (%)	NOACs, n (%)
Antiplatelet	280 (84.08)	53 (15.92)	8 (5.41)	140 (94.59)
non-antiplatelet	763 (84.78)	137 (15.22)	42 (10.32)	365 (89.68)
*χ* ^2^	0.090		3.197	
*p*	0.765		0.074	

Abbreviations: VKA, vitamin K anticoagulants; NOACs, Non-vitamin K antagonist oral anticoagulants; NCDP, national centralized drug procurement.

The utilization rate of NOACs increased significantly after NCDP, regardless of whether the patients with atrial fibrillation had antiplatelet or not, and the increase was statistically significant(*χ* ^2^ = 264.007、656.810, *p* < 0.001) as detailed in Table 3.

**TABLE 3:** Comparison of anticoagulants between antiplatelet and non-antiplatelet patients before and after NCDP.

Group	Antiplatelet		Non-antiplatelet	
	VKA, n (%)	NOACs, n (%)	VKA, n (%)	NOACs, n (%)
Before NCDP	280 (84.08)	53 (15.92)	763 (84.78)	137 (15.22)
After NCDP	8 (5.41)	140 (94.59)	42 (10.32)	365 (89.68)
*χ* ^2^	264.007		656.810	
*p*	**0.000**		**0.000**	

Abbreviations: VKA, vitamin K anticoagulants; NOACs, Non-vitamin K antagonist oral anticoagulants; NCDP, national centralized drug procurement; bold, *p* < 0.05).

### 3.4 Bleeding in patients before and after NCDP

**FIGURE 3:**
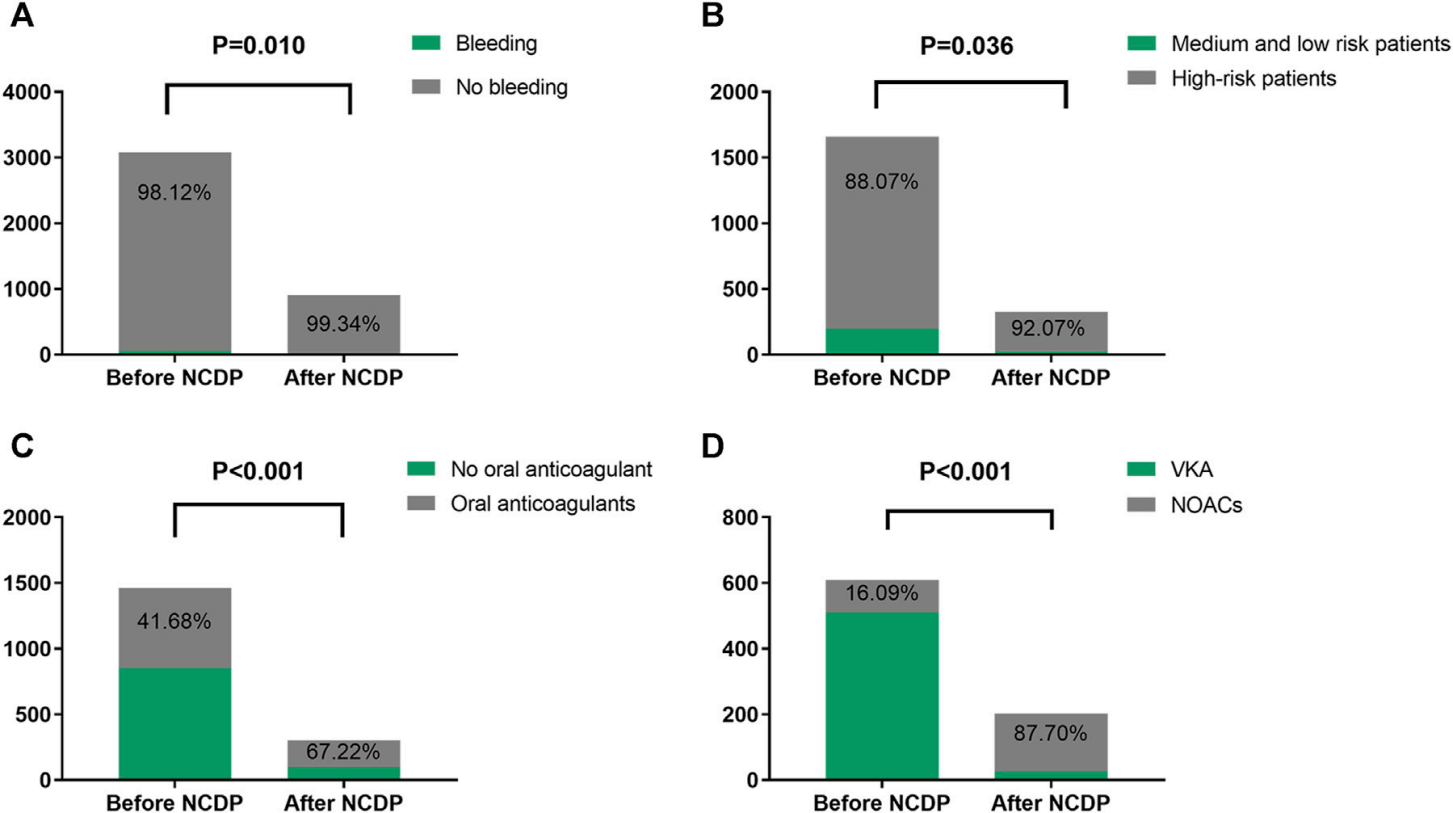
Comparison of bleeding events, high-risk patients with multiple hospitalizations, and drug use before and after NCDP. **(A)** Bleeding in patients before and after NCDP. **(B)** Comparison of high-risk patients before and after NCDP. **(C)** Comparison of anticoagulant use in high-risk patients before and after NCDP. **(D)** Comparison of NOACs use in high-risk patients before and after NCDP.

An examination of Figure 3A reveals that among the total patient cohort in the study, 64 individuals experienced hemorrhage events. Prior to the implementation of the NCDP policy, there were 58 cases (1.88%) of bleeding. Following the enactment of the policy, the number of hemorrhage events significantly reduced to 6 cases (0.66%). This substantial decrease in hemorrhage events post-NCDP policy is statistically significant (*χ* ^2^ = 6.585, *p* = 0.01), as detailed in Table 4.

**TABLE 4:** Bleeding and comparison of high-risk patients and drug use in patients before and after NCDP.

Group		Before NCDP	After NCDP	*χ* ^2^	*p*
ALL		3081	905		
Bleeding, n (%)		58 (1.88)	6 (0.66)	6.585	**0.010**
No bleeding, n (%)		3023 (98.12)	899 (99.34)		
Medium and low-risk patients, n (%)		198 (11.93)	26 (7.93)	4.398	**0.036**
High-risk patients, n (%)		1461 (88.07)	302 (92.07)		
High-risk patients	No oral anticoagulant, n (%)	852 (58.32)	99 (32.78)	62.851	**0.000**
	Oral anticoagulation, n (%)	609 (41.68)	203 (67.22)		
	VKA, n (%)	511 (83.91)	27 (13.30)	339.499	**0.000**
	NOACs, n (%)	98 (16.09)	176 (87.70)		

Abbreviations: VKA, vitamin K anticoagulants; NOACs, Non-vitamin K antagonist oral anticoagulants; NCDP, national centralized drug procurement; bold, *p* < 0.05).

Based on the analysis of bleeding subtypes and events before and after NCDP, it is concluded that there is no statistical difference in bleeding events and bleeding subtypes before and after the implementation of NCDP policy (*χ* ^2^ = 5.322、0.333, *p* > 0.05), as detailed in Table 5.

**TABLE 5:** Analysis of bleeding subtypes and bleeding events before and after NCDP.

Group		Before NCDP (n = 58)	After NCDP (n = 6)	χ^2^	*p*
major bleeding	gastrointestinal bleeding, n (%)	8 (13.79)	0 (0)	5.322	0.378
	Intracerebral hemorrhage, n (%)	5 (8.62)	0 (0)		
non-major bleeding	Hematuria, n (%)	15 (25.86)	3 (50.00)		
	Epistaxis, n (%)	13 (22.42)	2 (33.33)		
	gastrointestinal bleeding, n (%)	9 (15.52)	1 (16.67)		
	Skin bleeding, n (%)	8 (13.79)	0 (0)		
Bleeding event	major bleeding, n (%)	13 (22.41)	0 (0)	0.333	0.240
	non-major bleeding, n (%)	45 (77.59)	6 (100)		

Abbreviations: NCDP, national centralized drug procurement.

Univariate analysis identified gender, hypertension, stroke, age ≥65 years, antiplatelet drugs, concomitant use of anticoagulants and antiplatelets, hepatic and renal insufficiency, and the NCDP policy as relevant factors affecting bleeding in AF patients. Multivariate analysis further confirmed that hypertension [*OR* = 1.979, 95% *CI* (1.132, 3.462), *p* = 0.017], stroke [*OR* = 1.375, 95% *CI* (1.023, 1.847), *p* = 0.035], age ≥65 years [*OR* = 0.339, 95% *CI* (0.188, 0.612), *p* < 0.001], combination therapy of anticoagulants and antiplatelets [*OR* = 3.620, 95% *CI* (1.752, 7.480), *p* < 0.001], hepatic and renal insufficiency [*OR* = 4.294, 95% *CI* (2.28, 8.084), *p* < 0.001], and the NCDP policy [*OR* = 0.295, 95% *CI* (0.115, 0.753), *p* = 0.011] are significant risk factors for bleeding in patients with atrial fibrillation (Table 6).

**TABLE 6:** Logistic analysis of factors affecting bleeding in patients with atrial fibrillation.

Variables	Univariate analysis	*p*	Multivariate analysis	*p*
	*OR*(95% *CI*)		*OR*(95% *CI*)	
gender	0.553 (0.323–0.949)	**0.031**	0.679 (0.390–1.180)	0.170
heart failure	0.931 (0.554–1.566)	0.788		
hypertension	2.107 (1.229–3.612)	**0.007**	1.979(1.132-3.462)	**0.017**
type 2 diabetes	1.003 (0.490–2.052)	0.994		
stroke	1.367 (1.032–1.081)	**0.029**	1.375 (1.023–1.847)	**0.035**
Vascular lesion	1.600 (0.753–3.402)	0.222		
age ≥65 years	0.439 (0.250–0.771)	**0.004**	0.339 (0.188–0.612)	**0.000**
Anticoagulant	0.693 (0.404–1.189)	0.183		
Antiplatelet	1.761 (1.045–2.966)	**0.033**	0.708 (0.359–1.397)	0.319
Anticoagulation combined with antiplatelet	3.088 (1.740–5.480)	**0.000**	3.620 (1.752–7.480)	**0.000**
coronary artery disease	0.836 (0.497–1.406)	0.499		
hepatic and renal insufficiency	4.640 (2.578–8.353)	**0.000**	4.294 (2.281–8.084)	**0.000**
NCDP policy	0.317 (0.126–0.796)	**0.014**	0.295 (0.115–0.753)	**0.011**

Abbreviations: NCDP, national centralized drug procurement; OR, odds ratio; CI, confidence interval; bold, *p* < 0.05).

### 3.5 Effect of NCDP on patients with multiple hospitalizations

Out of the 3,986 patients in the study, 1,987 experienced multiple hospitalizations. Prior to the NCDP policy, 1,659 of these patients had repeated hospitalizations. Figure 3B shows that among the patients with recurrent hospitalizations before NCDP, 1,461 individuals (88.07%) were categorized as high-risk based on their CHA2DS2-VASC score. Post-NCDP, the number of high-risk patients increased marginally to 302 (92.07%), with 26 (7.93%) classified as low-risk. There were significant differences in the distribution of middle and low-risk patients between the pre-and post-NCDP periods (*χ* ^2^ = 4.398, *p* = 0.036).

According to Figure 3C, there was a notable shift in the use of oral anticoagulants among high-risk patients surrounding the NCDP policy. Before NCDP, 852 high-risk patients (58.32%) did not receive oral anticoagulants, whereas 609 (41.68%) were prescribed them. Post-NCDP, there was a rise in high-risk patients receiving oral anticoagulants, with 203 (67.22%) on these medications and 99 (32.78%) not receiving them. This increase in oral anticoagulant usage among high-risk AF patients was statistically significant (*χ* ^2^ = 62.851, *p* < 0.001).

Figure 3D indicates significant changes in the choice of oral anticoagulation therapy among high-risk patients before and after the NCDP policy. Initially, the majority, 511 patients (83.91%), were treated with VKA, and 98 (16.09%) with NOACs. Post-NCDP, there was a considerable shift, with 27 patients (13.30%) on VKA and 176 (87.70%) on NOACs. This dramatic increase in the proportion of high-risk patients receiving NOACs post-NCDP is noteworthy (*χ* ^2^ = 339.499, *p* < 0.001).

### 3.6 The impact of NCDP on re-hospitalization patients before and after the policy

**FIGURE 4:**
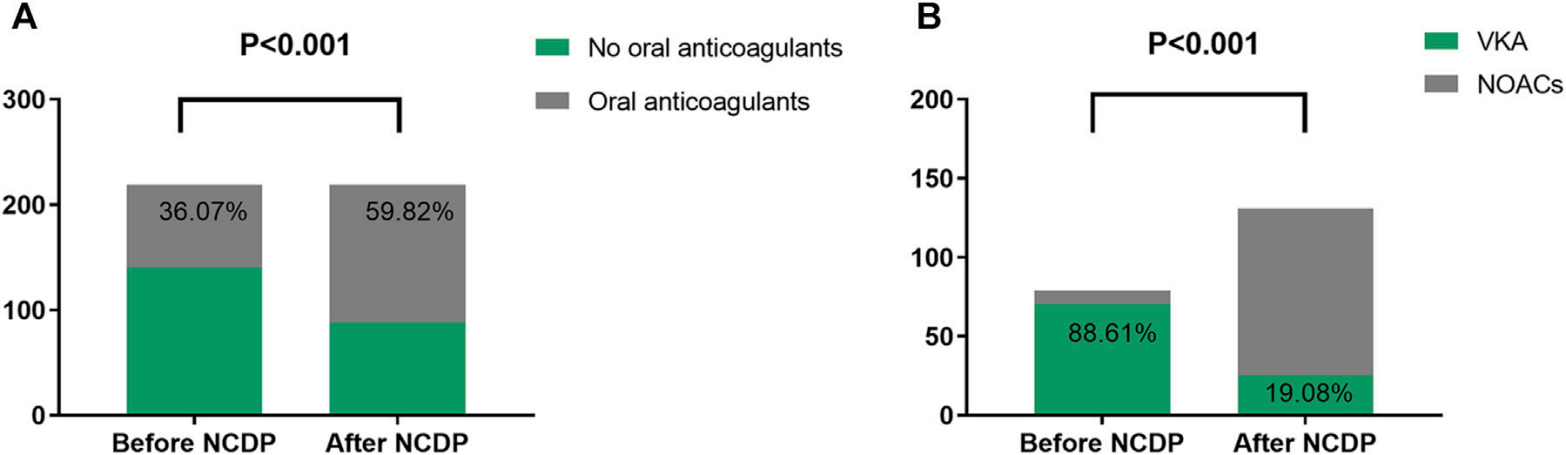
Analysis of anticoagulants **(A)** and NOACs **(B)** use in 219 patients hospitalized before and after NCDP.

Within the total cohort of 3,986 patients in this study, a subset of 1,987 experienced multiple hospitalizations. Of these, 219 patients had been hospitalized before the NCDP policy and required subsequent hospitalization post-policy implementation. Figure 4 offers an in-depth analysis of anticoagulant usage among these 219 patients, both before and after the NCDP policy. Initially, 140 patients (63.93%) declined oral anticoagulant therapy pre-NCDP, but post-policy, the number of patients refusing these medications decreased to 88 (40.18%). Conversely, the number of patients voluntarily taking anticoagulants increased from 79 (36.07%) before NCDP to 131 (59.82%) after its implementation, indicating a growing acceptance and willingness to use anticoagulant therapy. This rise in anticoagulant usage post-NCDP was statistically significant (*χ* ^2^ = 24.736, *p* < 0.001) (Table 7).

**TABLE 7:** Analysis of drug use in 219 patients hospitalized before and after NCDP.

Group	No oral anticoagulant, n (%)	Oral anticoagulation, n (%)	VKA, n (%)	NOACs, n (%)
Before NCDP	140 (63.93)	79 (36.07)	70 (88.61)	9 (11.39)
After NCDP	88 (40.18)	131 (59.82)	25 (19.08)	106 (80.92)
*χ* ^2^	24.736		96.153	
*p*	0.000		0.000	

Abbreviations: VKA, vitamin K anticoagulants; NOACs, Non-vitamin K antagonist oral anticoagulants; NCDP, national centralized drug procurement.

Before NCDP, the majority of patients, precisely 70 (88.61%), were treated with oral VKAs, while only 9 (11.39%) were prescribed NOACs. Post-NCDP, there was a noticeable change in the preference for oral anticoagulants: the number of patients on VKAs reduced to 25 (19.08%), while those on NOACs markedly increased to 106 (80.92%). It was observed that 91 patients specifically opted for NOACs post-NCDP, influenced by factors such as reduced prices and other considerations. The increase in NOAC utilization among the 219 patients with multiple hospitalizations post-NCDP was substantial (*χ* ^2^ = 96.153, *p* < 0.001) (Table 7).

### 3.7 Logistic univariate analysis of the factors affecting the change of anticoagulants in patients

With reference to previous studies, factors that may affect patients’ anticoagulant therapy changes were included. The data presented in Table 8 revealed that the implementation of the NCDP policy [*OR* = 28.223, 95% *CI* (13.148, 60.585), *p* < 0.001] and bleeding events [*OR* = 27.772, 95% *CI* (3.213, 240.026), *p* = 0.003] were significant factors affecting the alteration of anticoagulant medications in patients.

**TABLE 8:** Factors affecting dressing change in patients with AF.

Variables	Univariate analysis	*p*	Multivariate analysis	*p*
	*OR* (95% *CI*)		*OR* (95% *CI*)	
gender	1.357 (0.788–2.338)	0.271		
age	1.022 (0.993–1.051)	0.139		
Type of medical insurance	0.408 (0.077–2.148)	0.290		
Hospitalization expenses	0.853 (0.546–1.332)	0.483		
Antiplatelet	0.629 (0.369–1.073)	0.089		
Bleeding events or poor INR control	11.443 (1.439–91.013)	**0.021**	27.772 (3.213–240.026)	**0.003**
NCDP	23.512 (11.242–49.171)	**0.000**	28.223 (13.148–60.585)	**0.000**

Abbreviations: INR, international normalized ratio; OR, odds ratio; NCDP, national centralized drug procurement; bold, *p* < 0.05).

## 4 Discussion

This retrospective study, encompassing the period from 1 January 2017, to 30 June 2022, focused on evaluating the clinical and economic outcomes of 3,986 patients diagnosed with NVAF. The primary aim was to conduct a comparative analysis of patients’ general health, anticoagulant utilization, and the incidence of hemorrhagic events before and after the implementation of the NCDP policy.

Contrary to expectations, our findings indicate that the NCDP policy did not lead to a decrease in hospitalization expenses. Instead, we observed a significant increase in the average cost of hospitalization post-policy implementation. The average hospitalization expense was 8,900.57 ± 9,023.02 CNY before the NCDP, which escalated to 9,829.99 ± 10,886.87 CNY afterward. This increase, both considerable and statistically significant, suggests that the NCDP policy had a direct and substantial impact on the costs associated with hospitalization.

It is essential to contextualize these findings within the broader landscape of healthcare economics. Recent studies (Kwon et al., 2013; Kwon et al., 2019) have indicated that the mere reduction of drug prices does not automatically translate into a decrease in overall drug expenditures. This observation aligns with the concept of the “bypass effect” in drug policy, which posits that reductions in drug prices can be offset by other factors, negating the expected cost savings. In our search for additional studies conducted in Korea (Suh et al., 2018), we noted clear trends in antidiabetic drug spending. Overall expenditures dropped by 6%, with discounted drugs seeing a 23% reduction. However, after a new pricing policy, spending on drugs with fixed prices increased by 16% despite stable usage rates. This reflects a notable shift from discounted to regularly priced hypoglycemic drugs, hinting at higher treatment demands possibly driving up healthcare costs. Our study’s results corroborate this notion, suggesting that while the NCDP policy effectively reduced drug prices, its overall impact on hospitalization costs was paradoxically opposite, possibly due to other contributing factors in the healthcare system.

Our study has shed light on evolving trends in the choice of oral anticoagulants among patients with NVAF following the NCDP reform. Notably, there was a marked increase in the preference for oral anticoagulants post-NCDP. Pre-policy, around 40.02% of patients opted for oral anticoagulants, but this figure escalated to 61.33% after the policy’s implementation, indicating a substantial shift in patient preference towards these medications.

Additionally, our research highlights a significant surge in the usage of oral NOACs within the anticoagulant category. Prior to the NCDP, NOACs represented approximately 15.41% of the anticoagulant choice among patients. This proportion dramatically increased to 90.99% post-policy, suggesting a robust preference for NOACs over other anticoagulants. This trend reflects the NCDP policy’s influence in steering patient choices towards NOACs, marking them as a preferred treatment option.

Research, including studies by Barcellona (Barcellona et al., 1998), has indicated that inadequate control of anticoagulation with Vitamin K Antagonists (VKAs) can lead to increased bleeding complications. This underscores the significance of optimal anticoagulation management. NOACs offer several advantages over VKAs, notably the elimination of routine International Normalized Ratio (INR) monitoring due to their predictable pharmacokinetics and pharmacodynamics. The reduced need for frequent follow-ups when using NOACs is a considerable benefit. Despite the inherent bleeding risks associated with anticoagulant therapy, studies have consistently shown that NOACs are generally associated with a lower risk of bleeding compared to VKAs (Steffel et al., 2018).

European clinical guidelines have widely recognized the benefits of NOACs in stroke prevention, particularly in AF patients, including those newly diagnosed with AF. Many of these guidelines rate NOACs as a top-tier recommendation (I, A) (Kirchhof et al., 2016). The current guidelines from the European Society of Cardiology (ESC) advocate the use of NOACs in most patients, a recommendation substantiated by positive outcomes observed in large-scale clinical trials (Connolly et al., 2009; Granger et al., 2011; Patel et al., 2011; Giugliano et al., 2013).

The utilization of NOACs in certain regions, especially grassroots areas, was previously limited due to their high costs before the implementation of the NCDP policy. This financial barrier often impeded patients’ ability to maintain continuous drug compliance. The introduction of the NCDP policy has significantly transformed the landscape of anticoagulant therapy. By incorporating NOACs into the centralized procurement framework, their prices have been markedly reduced. This reduction has eased the financial burden on patients, enhancing the accessibility and affordability of NOACs. Consequently, there has been a considerable improvement in patient compliance with oral anticoagulant therapy. Patients are now more likely to adhere to their prescribed treatment regimens, free from the burden of high medication costs. This has resulted in an increased proportion of patients opting for oral anticoagulants, particularly NOACs, following the NCDP policy.

The study found that the NCDP policy did not significantly alter anticoagulant choices when used alongside antiplatelet therapy. However, a notable shift was observed in the increased preference for NOACs among atrial fibrillation patients receiving antiplatelet therapy post-NCDP, rising from 15.92% to 94.59%. This suggests that healthcare providers are increasingly favoring NOACs over VKAs when antiplatelet therapy is necessary, likely due to NOACs’ advantages, such as simplified dosing, reduced bleeding risk, and no need for routine INR monitoring.

For patients undergoing antiplatelet therapy for acute coronary syndrome (ACS), there was no significant variation in anticoagulant usage, irrespective of the NCDP policy. This uniformity may be attributed to grassroots doctors adhering to established guidelines. Previous studies (Giugliano et al., 2013) have shown that combining VKA with dual antiplatelet therapy greatly increases bleeding risk in ACS patients. International guidelines (Brignole et al., 2013; January et al., 2014) recommend NOACs as the preferred anticoagulation option for managing AF patients with concurrent coronary heart disease (CHD). The 2020 ESC/EACTS guidelines (Hindricks et al., 2020) on AF diagnosis and management provide specific recommendations for managing AF patients with coexisting ACS, chronic coronary syndrome (CCS), or percutaneous coronary intervention (PCI). In cases where AF patients with ACS, CCS, or PCI meet the criteria for anticoagulation therapy with NOACs, the guidelines strongly advocate using NOACs alongside antiplatelet aggregation drugs (I, A).

In our cohort of 219 patients hospitalized before and readmitted after the NCDP policy, we observed significant changes in oral anticoagulant usage. The number of patients receiving oral anticoagulants increased from 79 to 131, while those not on any oral anticoagulant therapy decreased from 140 to 88, both pre-and post-NCDP, respectively. This trend indicated a notable rise in anticoagulant use among patients who experienced multiple hospitalizations before and after the implementation of the NCDP. Statistical analysis affirmed this trend, showing a marked difference in usage patterns.

Prior to the NCDP policy, 70 patients had chosen VKAs for their anticoagulant therapy. Post-policy implementation, however, there was a significant decline in the use of VKAs, with only 25 patients continuing this treatment. Conversely, the number of patients opting for NOACs surged to 106. Notably, 91 of these patients voluntarily switched to NOACs, influenced by the reduced prices. This shift towards NOACs, facilitated by their improved accessibility and affordability post-policy, was substantiated by statistical analysis, which demonstrated a significant increase in NOAC utilization after the NCDP. The difference in NOAC utilization rates before and after the policy was statistically significant.

Consistent with existing literature (Beyer-Westendorf et al., 2016; Hernandez et al., 2017; Jackevicius et al., 2017; Lamberts et al., 2017; Manzoor et al., 2017), our findings indicate that the discontinuation rate for NOACs is notably lower than for VKAs. The policy intervention led to a significant uptick in NOAC use, while VKA usage saw a declining trend. This preference for NOACs over VKAs aligns with their favorable profile in terms of efficacy, safety, convenience, and patient satisfaction.

These observations underscore the profound impact of healthcare policies on medication choices and patient compliance, particularly in the context of long-term treatment regimens. The NCDP policy has evidently played a pivotal role in shaping these trends, highlighting the importance of policy interventions in healthcare.

The analysis demonstrated a marked decrease in hemorrhagic events following the NCDP policy, with the rate falling from 1.88% to 0.66%, highlighting its effectiveness in enhancing the safety of anticoagulant therapy. A key challenge with VKAs like warfarin is the necessity for regular monitoring of blood coagulation function, typically through INR testing. For grassroots patients, maintaining INR within the therapeutic range of 2.0–3.0 can pose considerable difficulties, often leading to frequent outpatient visits or emergency department admissions due to complications from organ bleeding caused by VKA therapy. This can result in repeated bleeding episodes and poor medication adherence.

In contrast to VKAs, NOACs do not require routine coagulation parameter monitoring, which simplifies the treatment process. NOACs mitigate the limitations associated with VKAs and offer several benefits that enhance the safety and convenience of antithrombotic treatment. Numerous studies (Giugliano et al., 2016; Wilson et al., 2017; Inohara et al., 2018) have consistently shown that, compared to VKAs, NOACs are associated with a lower risk of intracranial hemorrhage and life-threatening bleeding. Additionally, patients on NOAC therapy generally have a more favorable prognosis in the event of severe hemorrhage (particularly extracranial hemorrhage) compared to those treated with VKAs. These findings hold significant implications for clinical decision-making, highlighting the preference for NOACs over VKAs in the management of patients.

Our logistic univariate analysis aimed to identify factors influencing the change in anticoagulant therapy. We discovered that age, type of medical insurance, hospitalization cost, and antiplatelet medication did not significantly impact the decision to change anticoagulant therapy. However, the NCDP policy emerged as the primary influencing factor. This outcome can be attributed to the policy’s role in reducing the cost of NOACs. Coupled with this price reduction, NOACs offer several advantages over traditional anticoagulants, including fewer side effects and the elimination of routine INR monitoring. These benefits, combined with clinician recommendations and advice from patients’ social circles, likely led to a marked increase in individuals choosing oral NOACs as their preferred anticoagulant therapy.

## 5 Limitations

One of the primary limitations of this study is its exclusive focus on medication usage among inpatients within a general hospital setting. This approach may not fully capture the broader spectrum of NVAF management, which includes outpatient care and treatment in various medical specialties. Future research should aim to incorporate a more comprehensive dataset, potentially encompassing a wider range of healthcare settings and long-term patient outcomes, to provide a more holistic understanding of NVAF management.

## 6 Conclusion

Our study provides robust evidence highlighting the substantial impact of the NCDP policy in enhancing anticoagulation management among patients with AF. Notably, the policy has been instrumental in improving medication adherence, facilitating the adoption of more effective anticoagulation strategies, and significantly reducing the incidence of hemorrhagic complications in this patient population. Moreover, the NCDP policy has emerged as a crucial factor influencing the decision-making process of healthcare providers and patients, particularly in transitioning to more preferable anticoagulant therapies such as NOACs. This shift underscores the policy’s effectiveness in not only reducing medication costs but also in potentially transforming treatment paradigms for AF patients.

## Data Availability

The raw data supporting the conclusion of this article will be made available by the authors, without undue reservation.

## Abbreviations

NCDP, National Centralized Drug Procurement; NOACs, non-vitamin K antagonist oral anticoagulants; NVAF, non-valvular atrial fibrillation; AF, Atrial fibrillation; VKA, vitamin K anticoagulants; CNSSS, China Stroke Screening Program; ESC, European Society of Cardiology; EACTS, European Association for Cardio-Thoracic Surgery; CNY, Chinese Yuan; ACS, acute coronary syndrome; CHD, coronary heart disease; CCS, chronic coronary syndrome; PCI, percutaneous corocnary intervention.
